# Inositol phosphate kinases in the eukaryote landscape

**DOI:** 10.1016/j.jbior.2020.100782

**Published:** 2021-01

**Authors:** Debabrata Laha, Paloma Portela-Torres, Yann Desfougères, Adolfo Saiardi

**Affiliations:** Medical Research Council Laboratory for Molecular Cell Biology, University College London, WC1E6BT, London, UK

**Keywords:** Inositol, Metabolism, Evolution, Kinase, Plant, Animal, Signalling, Phytic acid

## Abstract

Inositol phosphate encompasses a large multifaceted family of signalling molecules that originate from the combinatorial attachment of phosphate groups to the inositol ring. To date, four distinct inositol kinases have been identified, namely, IPK, ITPK, IPPK (IP5–2K), and PPIP5K. Although, ITPKs have recently been identified in archaea, eukaryotes have taken advantage of these enzymes to create a sophisticated signalling network based on inositol phosphates. However, it remains largely elusive what fundamental biochemical principles control the signalling cascade. Here, we present an evolutionary approach to understand the development of the ‘inositol phosphate code’ in eukaryotes. Distribution analyses of these four inositol kinase groups throughout the eukaryotic landscape reveal the loss of either ITPK, or of PPIP5K proteins in several species. Surprisingly, the loss of IPPK, an enzyme thought to catalyse the rate limiting step of IP_6_ (phytic acid) synthesis, was also recorded. Furthermore, this study highlights a noteworthy difference between animal (metazoan) and plant (archaeplastida) lineages. While metazoan appears to have a substantial amplification of IPK enzymes, archaeplastida genomes show a considerable increase in ITPK members. Differential evolution of IPK and ITPK between plant and animal lineage is likely reflective of converging functional adaptation of these two types of inositol kinases. Since, the IPK family comprises three sub-types IPMK, IP6K, and IP3–3K each with dedicated enzymatic specificity in metazoan, we propose that the amplified ITPK group in plant could be classified in sub-types with distinct enzymology.

## 1 Introduction

Living organisms took advantage of the metabolic stability and ease of synthesis of inositol not only to use it as osmolyte (Novak et al., 1999), but also to create a vast family of intracellular messengers including the lipid-bound phosphoinositides (PIPs) and the soluble inositol phosphates (IPs). The combinatorial attachment of phosphate moieties to the six hydroxyl groups of inositol could yield up to 63 different IPs (York, 2006) of which I (1,4,5)P_3_, the calcium releasing factor is the most well-known. The molecular complexity of IPs signalling network is further augmented with the possibility to have pyro-phosphate group(s) attached to the inositol ring (Shah et al., 2017; Wilson et al., 2013) such as in IP_7_ and IP_8_, referred to as inositol pyrophosphates. These highly polar and energy-rich species occupy the center stage of IPs research due to their key role in regulating a large array of fundamental cellular functions including energy metabolism, phosphate homeostasis and immune responses in yeasts, metazoans and plants (Azevedo and Saiardi, 2017; Dong et al., 2019; Laha et al., 2015; Li et al., 2020; Lopez-Sanchez et al., 2020; Wilson et al., 2019; Zhu et al., 2019). The intricacy of this signalling network is further highlighted by several protein structures that unexpectedly reveal the presence of IPs in their crystal (Blind, 2020). The metabolic complexity of IPs network and the absence of a chromophore facilitating their detection are the major challenges associated with studying the cellular function of these molecules. Therefore, a major focus in the field is to establish novel methods allowing us to map the subcellular concentration as well as the molecular identity of different IPs (Qiu et al., 2020; Harmel et al., 2019; Ito et al., 2018; Losito et al., 2009; Wilson et al., 2015).

IPs are synthesized by four distinct enzyme (kinase) families which are thought to be widespread across the eukaryotes. These kinase classes are defined by specific sequence signature, and annotated in Pfam database with specific entries. Among the four types, inositol polyphosphate kinase (IPK, PF03770) is the most characterised one and could be further categorized into three distinct subgroups, the I (1,4,5)P_3_-3 kinase (IP3–3K), the inositol phosphate multikinase (IPMK, Arg82 or Ipk2) and the inositol hexakisphosphate kinase (IP6K, Kcs1) (Shears and Wang, 2019). The remaining three inositol kinase families are the inositol pentakisphosphate 2-kinase (IPPK, IP5–2K or Ipk1: PF06090) (Gonzalez et al., 2010); the inositol 1,3,4-trisphosphate 5/6-kinase (ITPK1, PF05770) (Miller et al., 2005); and the diphosphoinositol pentakisphosphate kinase (PPIP5K, Vip1 or VIH). The latter kinase usually possesses an interesting dual-domain structure with a N-terminal kinase region (PF18086) and a C-terminal histidine phosphatase superfamily domain (PF00328) present in most phytases and acid phosphatase proteins (Dollins et al., 2020; Randall et al., 2020).

The recent identification of ITPK kinases in Lokiarchaeota genome, a novel archaea phylum that might have contributed to the eukaryogenesis, challenges the notion of a restricted emergence of IP synthesis pathways in the eukaryotic lineage. Identification of this kinase family in Lokiarchaeota has also permitted to define additional IP substrates of this enzyme. Remarkably, this has allowed to uncover the enzymology responsible for the cytosolic, PLC independent, route of IPs synthesis (Desfougeres et al., 2019).

Our biochemical understanding of IP-kinases is instrumental in deciphering the physiological functions of different IPs (Shears, 2004). For instance, the heterologous expression of eukaryote IP kinases in conjunction with PIP kinases and phospholipase in bacteria could reconstitute the IPs metabolic network and thus creates a simplified model to dissect IPs metabolism (Clarke et al., 2019). Similarly, many information about IPs function are gained by defining the cell biology pathways controlled by different IP kinases. The knockdown, knockout or pharmacologically blocking these kinases have revealed a pleiotropic effect on eukaryote cell physiology (Burton et al., 2013; Chanduri et al., 2016; Laha et al., 2015; Rao et al., 2014; Sahu et al., 2020; Zhu et al., 2017). Both the complexity of the IPs metabolic network and the elaborated informational flux occurring by converting one IP to another require additional insight to appreciate this signalling network. To this aim, we will undertake an evolutionary approach providing us new perspective to further dissect the physiological roles of different IPs. Studying the distribution of IP-kinases across the evolutionary tree will grant key insights to be incorporated towards a comprehensive understanding of the IPs signalling network.

## 2 Materials and methods

### 2.1 Identification of inositol phosphate kinase

Two criteria were employed to select the organisms to be included in this study: we selected species representative of the different eukaryotic subkingdoms and those of organisms used as experimental models. The list of analysed organisms and their corresponding taxonomy ID is depicted in Table 1. The collected sequences are reflective of databases accessed in August 2019 and revisited in April 2020. Protein sequences were manually curated and cross-referenced to obtain only one representative protein isoform per gene. Collection of candidate IP kinase sequences across eukaryotes was carried out through a combination of methods. Initially, we performed a Hidden Markov Model (HMM) searches (Potter et al., 2018) against the UniProtKB database using the HMM models for all four enzymes available from Pfam (IPK: PF03770.16, PPIP5K: PF18086.1, ITPK1: PF17927.1, ITPK1: PF05770.11 and IPPK: PF06090.12). Default 0.01 sequence threshold and 0.03 hit threshold were used for HMM searches using the HMMER biosequence analysis tool (https://www.ebi.ac.uk/Tools/hmmer/). We also performed Protein Basic Local Alignment Search Tool (BLAST) searches using IP-kinase members found in *Saccharomyces cerevisiae* and *Homo sapiens*. A permissive cut-off e-value of 0.01 (Pearson, 2013) was employed given the high sequence diversity of kinase member of these families. HMM and BLAST searches were performed by restricting the search to the taxonomy of the selected organisms. The results from HMM and BLAST searches of each organism were compared and combined.

**Table 1:** Inositol phosphate kinase members across eukaryote species.

Domain	Sub-Kingdom	TaxID	Species	IPK	IPPK	ITPK1	PPIP5K
Eukaryota	Opisthokonta	6239	*Caenorhabditis elegans*	5	1	0	1
		5207	Cryptococcus neoformans	3	1	0	1
		7955	*Danio rerio*	10	1	2	3
		7227	*Drosophila melanogaster*	3	1	0	1
		9606	*Homo sapiens*	7	1	1	2
		6087	*Hydra vulgaris*	5	1	3	3
		10,090	*Mus musculus*	7	1	1	2
		4932	*Saccharomyces cerevisiae*	2	1	0	1
		4896	*Schizosaccharomyces pombe*	2	1	0	1
	Amoedozoa	44,689	*Dictyostelium discoideum*	4	1	1	1
		5759	Entamoeba histolytica	4	0	4	0
		13,642	Heterostelium pallidum	4	1	1	1
	Archaeplastida	3702	*Arabidopsis thaliana*	2	4	4	2
		3055	Chlamydomonas reinhardtii	1	2	3	2
		3197	Marchantia polymorpha	1	2	2	1
		4530	Oryza sativa	1	1	6	2
	SAR	353,152	Cryptosporidium parvum	1	0	0	1
		1,519,565	Fistulifera solaris	2	1	2	2
		2,315,210	Hondaea fermentalgiana	2	1	0	1
		403,677	Phytophthora infestans	1	1	2	2
		37,360	Plasmodiophora brassicae	1	1	0	1
		5833	Plasmodium falciparum	2	0	0	1
		46,433	Reticulomyxa filosa	1	1	3	1
		5911	Tetrahymena thermophila	2	0	1	1
	Excavata	5741	Giardia intestinalis	1	0	0	1
		5664	Leishmania major	2	1	1	0
		5722	Trichomonas vaginalis	8	1	2	0
		5691	Trypanosoma brucei	2	1	1	0
	Hacrobia	2903	Emiliania huxleyi	5	0	1	5
		55,529	Guillardia theta	3	1	1	1

Collected sequence are available upon request.

### 2.2 Identification of IP kinase across the eukaryotic landscape

To identify the possible loss of specific IP kinases throughout the eukaryotic kingdom, we screened the reference proteomes of the organisms whose genomes have been completely sequenced, thus providing a comprehensive coverage of species across the tree of life. While this approach limited the number of species to be analysed, it prevented the detection of a kinase absence due to incomplete genome sequencing. The absence of different kinases among UniProt eukaryotic reference proteomes (https://www.uniprot.org/proteomes) was identified by annotating organisms with eukaryotic proteomes based on the presence or absence of different InterPro kinase family members.

This analysis was restricted to IPK, ITPK1 and IP5–2K, given the availability of their InterPro family entries (IPK: IPR005522, ITPK1: IPR008656 and IP5–2K: IPR009286). PPIP5K was not included in this analysis due to the absence of an annotated InterPro kinase family for the kinase domain of this enzyme. The phosphatase domain of this dual-domain enzyme does have an Interpro entry but the screening with this domain will collect mainly phosphatases.

Those eukaryotic proteomes identified by a proteome identifier (UPID, e.g.: UP000000000) that did not have an InterPro entry for either of the three inositol phosphate kinases were annotated. Individual absences in the species corresponding to the proteome identifiers were collected.

### 2.3 Phylogenetic tree

A tree of reference eukaryotic proteomes was generated and branches in which all species of a given taxon had the same kinase absence were annotated as bona fide absences across the whole taxon. Other taxa without complete loss of a kinase family in all constituent species were collapsed and left uncoloured. The results were displayed in a tree of eukaryotic species with reference proteomes generated through the NCBI Taxonomy tool Common Tree (https://www.ncbi.nlm.nih.gov/Taxonomy/CommonTree/wwwcmt.cgi). The final figure was obtained by manually annotating the tree using the FigTree software (http://tree.bio.ed.ac.uk/software/figtree/). An evolutionary tree with branch lengths proportional to divergence time was generated using TimeTree (http://timetree.org/) (Institute for Genomics and Evolutionary Medicine Center of Biodiversity, Temple University). The generated Newick file was used to generate the tree in the figure using FigTree. Species falling outside the tool coverage were manually added adjacent to those of the same taxa as described by the Open Tree of Life reference taxonomy (https://tree.opentreeoflife.org/opentree/argus/opentree12.3@ott93302). Inositol phosphate kinase numbers as described in Table 1 were manually annotated and colour coded for each species.

To generate plant ITPK phylogenetic tree the collected proteins sequences of Marchantia, Oryza, Arabidopsis and human were aligned using COBALT Constraint-based Multiple Alignment Tool) software (https://www.ncbi.nlm.nih.gov/tools/cobalt/cobalt.cgi) and the circular tree of fast minimal evolution using Kimura’ s neutral theory distance model was generated.

## 3 Results and discussion

### 3.1 Inositol metabolism and inositol phosphate kinases

Most multicellular eukaryotic cells are able to acquire inositol exogenously as ‘food’ from the extracellular environment. Furthermore, the majority of eukaryotes have the ability to synthesize it endogenously from glucose-6P. Thanks to its isomerization to inositol-3P carried out by the inositol phosphate synthase (IPS) called ISYNA1 in human or Ino1 in yeast (Desfougeres et al., 2019; Donahue and Henry, 1981). However, recent findings demonstrating the ability of isyna1^−/−^ null cell to synthesize inositol suggest the existence of ISYNA1 independent pathway of inositol synthesis (Qiu et al., 2020).

**Fig. 1:**
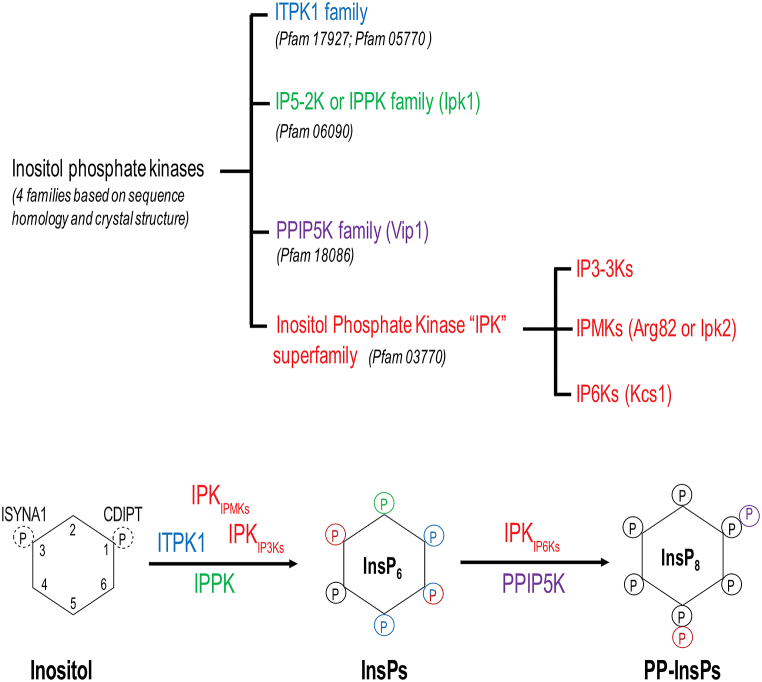
Inositol phosphate kinase families.

We will briefly highlight the fate of the two sources of cellular inositol since it will give insight on phosphorylation events that are independent of IP kinases. While endogenously synthesized inositol-3P could directly act as a substrate for ITPK1 (Desfougeres et al., 2019), inositol per se, thus the exogenously acquired inositol, is not a substrate for any of the four IP kinase families subject of our study (Fig. 1). Inositol instead could be phosphorylated by inositol-kinases, proteins unrelated to any of the IP kinase families. Inositol-kinases (also known as myo-inositol kinase) are present in archaeplastida (Shi et al., 2005) and in the amoeba *Dictyostelium discoideum* (Stephens et al., 1990) but are absent in metazoan (animal). Therefore, in animal, the only possibility for the exogenously acquired inositol to become an IP is to be substrate of phosphatidylinositol synthase (PIS or CDIPT) and be converted first in the lipid phosphatidylinositol. Conversely, in amoeba where inositol-kinases are present, exogenously acquired inositol could enter both IP synthetic pathways-the lipid dependent and the cytosolic routes although the latter is the preferred one (Drayer et al., 1994; Pisani et al., 2014).

Two main catalytic activities could be assigned to IP kinases. These enzymes could catalyse phosphorylation reaction thus generating phosphoester bond or could synthesize pyrophosphate moiety through the formation of a high-energy phosphoanhydride bond (Fig. 1). Enzymes catalysing the formation of phosphoester bond to the different positions of inositol ring include IPMK, ITPK1, IP3–3K and IPPK (IP5–2K); whereas enzymes generating high-energy phosphoanhydride bond are IP6K and PPIP5K.

The canonical phosphorylation and pyrophosphorylation positions for these enzymes are displayed in Fig. 1. It is important to highlight that these accredited phosphorylation and pyrophosphorylation positions have been established by studies employing mainly recombinant enzymes of mammalian or of yeast origin. It is plausible that IP kinase from different species might have acquired different position specificity. Many of these inositol phosphate kinases possess substrate promiscuity. For instance, the IP6K could use both IP_6_ and IP_5_ as substrates, while the two ‘multikinases’ IPMK and ITPK1 could phosphorylate at different positions of inositol ring and are able to recognize a vast array of IP substrates (Miller et al., 2005; Wang and Shears, 2017). Although Fig. 1 reveals that the ‘canonical’ kinase responsible for phosphorylation at position four (C4) is currently undefined, it is likely that one of the two multikinases, if not both, could phosphorylate this position at inositol ring. Alternatively, a new class of enzyme could be responsible for this phosphorylation.

Classifying an inositol phosphate kinase family as a phosphorylating or pyro-phosphorylating enzyme is not always straightforward. In fact, some member of plant ITPKs are also able to synthesize phosphoanhydride bond converting IP_6_ to IP_7_ and thus functioning as the ‘IP6K’ enzymes missing in the plant lineage (see below) (Laha et al., 2019). Similarly, mammalian and yeast IPMK are able to synthesize pyrophosphate species at least in vitro (Saiardi et al., 2001; Zhang et al., 2001).

This overview of the known inositol phosphate kinases reveals the presence of four distinct classes of enzymes showing substrate promiscuity, and flexibility on phosphorylation site. Evolution has certainly operated on this highly malleable system. This creates the platform to gain new insights on inositol phosphate signalling by studying the distribution of these four families of IP- kinases throughout eukaryote.

### 3.2 Absence of inositol phosphate kinases across eukaryotes

To gain insight into the distribution of inositol phosphate kinase families throughout the eukaryotic landscape, we examined the presence of these enzymes using BLAST homology searches against the selected genome and Hidden Markov Model (HMM) searches against the UniProtKB database as detailed in the material and methods. We searched for the inositol phosphate kinases in the organisms that are representative of the diverse eukaryotic subkingdoms and ideally are employed as laboratory experimental models. Table 1 depicts the representative species analysed along with the respective scoring for the presence of IPK, IPPK, ITPK1 and PPIP5K family members in their genomes. Notably, members of certain taxa showed patterns of absence of certain kinases. In order to determine with precision the origin of these kinase absences, an approach relying on whole proteomes was employed.

**Fig. 2:**
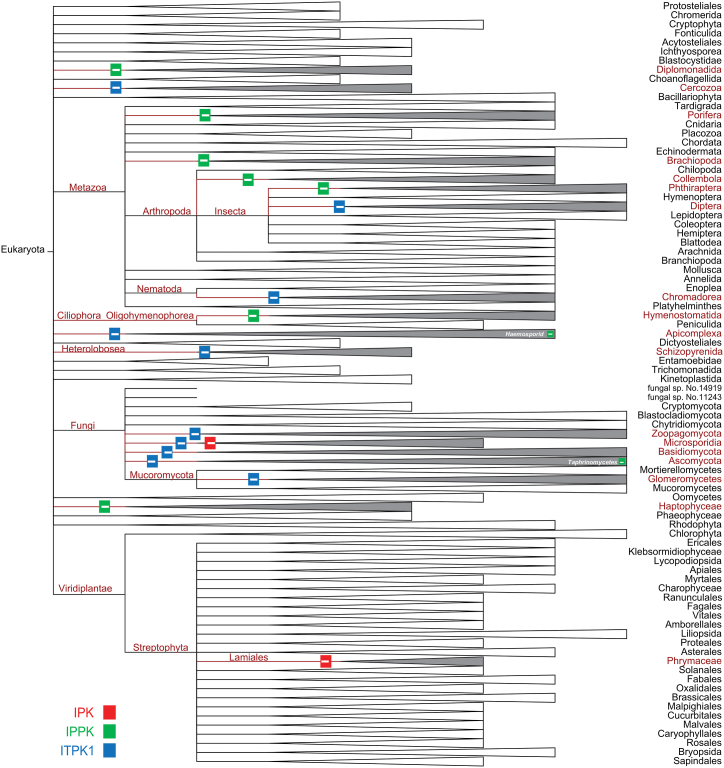
Inositol phosphate kinase absence across eukaryotic reference proteomes.

To assess the absence of certain inositol phosphate kinases in eukaryotes, reference eukaryotic proteomes were analysed. The absence of different kinases was identified by collecting InterPro family entries restricting our search to reference proteomes (see material and methods). The inositol phosphate kinase absence at the reference proteome level would provide reliable information on the bona fide nature of such an absence and not due to a partially sequenced genome. The analysis of specific inositol phosphate kinase absences was performed at the level of species and traced back to the eukaryotic tree of life for identification of taxa with a total loss of a particular inositol phosphate kinase. A colour-coded and condensed tree depicting IPK, IPPK and ITPK1 kinase absence across NCBI taxonomy database is represented in Fig. 2.

From this analysis, it becomes evident that certain generalisations previously made about the presence or absence of inositol phosphate kinases did not apply to the realm of complexity of eukaryotic taxonomy. Fungi, mainly thanks to work carried in the yeast *S. cerevisiae*, *S. pombe* and *C. neoformans* (Saiardi et al., 2018), had been believed to lack ITPK1 enzymes. However, this analysis unravelled that only a fraction of species in this taxon has a loss of this kinase. Although not complete, the ITPK1 loss observed in many fungal species, could be attributed to a general kinase, including protein kinase, loss in this taxon (Goldberg et al., 2006). The reduction of genome size in this taxon, closely related to metazoan, could underpin this phenomenon.

A frequent loss of IPPK (IP_5_–2K) was not previously perceived and its relevance lies in the indispensable function of this enzyme in the synthesis of IP_6_. The I (1,3,4,5,6)P_5_ two-kinase synthesizes IP_6_ by catalysis what could be considered the final step, perhaps the rate-limiting step, phosphorylation at the 2 hydroxyl (2-OH) of the inositol pentakisphosphate I (1,3,4,5,6)P_5_. One would expect that this enzyme might be required in all eukaryotes to produce IP_6_ and the derivatives, inositol pyrophosphate species. This seems not to be the case and points towards the potential catalytic flexibility of other inositol phosphate kinase(s) overcoming IPPK absence or to the existence of a novel 2-OH kinase that evolved independently outside the four major IP kinase families. More tantalising we could speculate on the existence of organisms lacking IP_6_ where IP_5_ might have taken over IP_6_ functionalities. To demonstrate this possibility, inositol polyphosphate profiling of the species lacking IPPK will be required. It is also possible that some organism could directly absorb IP_6_ as ‘food’ making IPPK redundant (see below).

This analysis reveals the absence of IPK family members only in one plant species Erythranthe guttata, the yellow monkeyflower, out of the 96 plant reference proteomes analysed. The vast majority of plants do not show the absence of the four major kinase families highlighting the functional relevance of these enzymes to synthesize inositol phosphates in such organisms. Emerging evidences support this view, and our understanding of inositol phosphate synthesis in plants is beginning to flourish (Kuo et al., 2018; Laha et al., 2015, 2019; Whitfield et al., 2020).

### 3.3 Distribution of inositol phosphates kinase in the tree of life

**Fig. 3:**
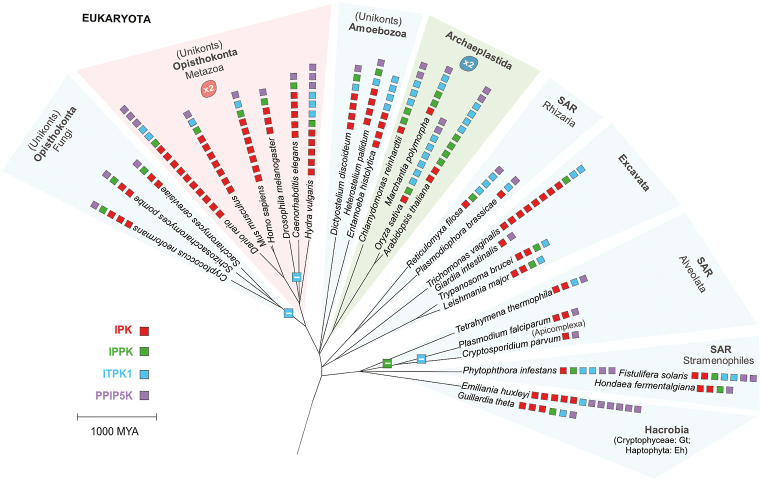
Inositol phosphate kinase members across the tree of life.

Beside absence, frequent cases of IP kinase amplification were detected from the quantitative analysis of the species in this study (Table 1). The taxonomic distributions of the presence, absence and amplification of a specific inositol phosphate kinase family are easier to interpret by generating a tree of life diagram (Fig. 3).

This analysis reveals that IPPK kinase is often present as a single copy in eukaryotes except in archaeplastida where multiple copies could exist. This could be attributed to the fundamental role of IP_6_ in controlling critical plant processes and to the not fully understood yet needs to synthesize excess of IP_6_ (phytate) accumulating into the vacuole (Nagy et al., 2009). In many species of Alveolata, which are primarily single-celled organisms, we observe a loss of IPPK. This could be consequences of some of their preferred modes of nutrition: predation and intracellular parasitism. The acquisition of IP_6_ would make redundant with its cellular synthesis and thus might result in IPPK loss. One remarkable example of taxa in Alveolata which shows both IPPK and ITPK kinase loss is Apicomplexa. Species in this taxon are obligate parasites and also show an ITPK loss, reflecting the tendency for loss of inositol phosphate kinases in the case of parasitism. This implies that the IPPK and ITPK-dependent IP species could be obtained from the host.

From the diagrammatic analysis (Fig. 3), amplification of the IPK family is evident in Metazoa. The complexity of these organisms illustrates towards a need for functional specialization of the IPK members. This was accomplished by catalytic diversification of IPK family which are categorized into subfamilies: the inositol polyphosphate multikinase IPMK, the inositol hexakisphosphate kinase IP6K and the IP3–3K that arise late in evolution in conjunction with IP_3_ driven calcium signalling in metazoan (Schell, 2010). Conversely, members of Archaeplastida show a considerable amplification in ITPK type of kinases. The ITPK augmentation could be simply attributed to the increased ploidy, a common speciation mechanism of plant. However, the increases in ploidy are not accompanied by amplification of IPK members of which a single gene usually is present in plant genomes. Therefore, a selective amplification of the ITPK family with a parallel reduction or constrain on the number of IPK has occurred in the plant lineage.

**Fig. 4:**
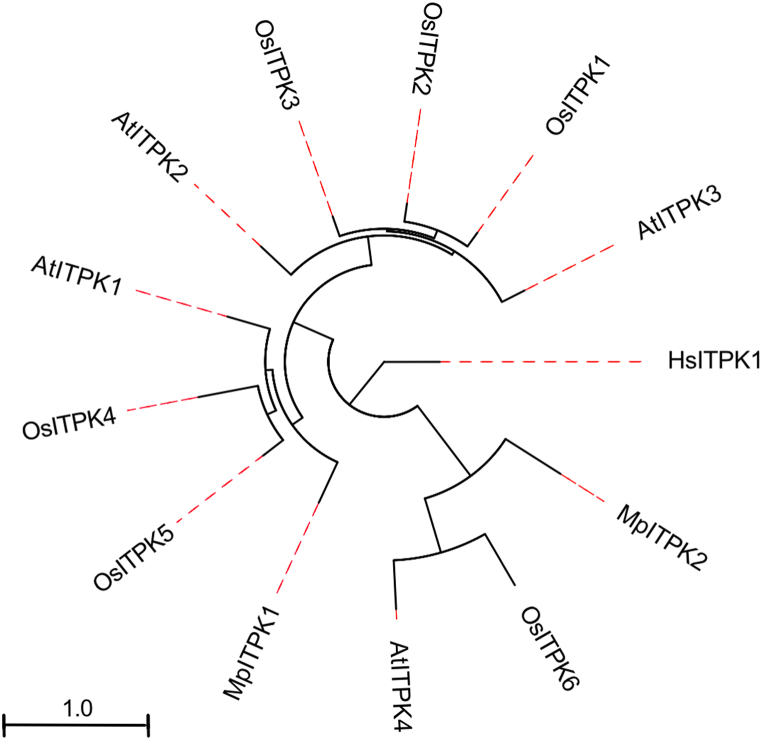
Phylogenetic analysis of plant ITPK protein.

Plant ITPK enzymes were originally identified as one of the kinase responsible for phytic acid synthesis in developing maize seeds (Shi et al., 2003). Compared to animal lineage, plant genome encodes a large number of ITPK proteins. For instance, a total of 6 ITPKs have been identified in rice (Oryza sativa) (Josefsen et al., 2007; Laha et al., 2019). *Arabidopsis thaliana* genome encodes 4 ITPK members ITPK1-4 (Kuo et al., 2018; Laha et al., 2019; Sweetman et al., 2007). At least 2 ITPK isoforms could be identified in the liverwort, Marchantia polymorpha (Fig. 3). The large number of IPK proteins in metazoan and the diversification of this family in three subfamilies strongly suggest for an analogous fate of the high number of ITPK1 family members in plant. To testify this hypothesis, we generated a phylogenetic tree of plant ITPKs (Fig. 4). This analysis revealed that Arabidopsis ITPK4 and rice ITPK6 are the outliers of ITPKs family and could be clustered in one separate group along with Marchantia ITPK2. In higher plants, ITPKs could be further subdivided in two groups. This phylogenetic study is supportive of a thorough parallel biochemical characterization of plant ITPK family members.

Biochemical and physiological functions of plant ITPKs are poorly investigated and thus remain largely elusive. In vitro studies have focused to replicate the ability of plant ITPKs to use I (1,3,4)P_3_ as a substrate. This is an activity carried out by all four Arabidopsis ITPK1-4 enzymes. However, plant ITPK1 can use a range of IP_3_ and IP_4_ substrates while ITPK4 possess IP_4_ isomerase activity (Stiles et al., 2008; Sweetman et al., 2007; Whitfield et al., 2020). The characterization of ITPK4-deficient plants suggests that this enzyme targets different yet uncharacterized inositol phosphate substrates in vivo (Parvin Laha et al., 2020; Kuo et al., 2018). Therefore, future work awaits to identify the in vivo target(s) of ITPK4 which would be instrumental to understand its mechanism of action. Tantalising, is the recent demonstration that Arabidopsis ITPK1-2 are able to convert IP_6_ to IP_7_ in vitro (Adepoju et al., 2019; Laha et al., 2019) and in vivo (Parvin Laha et al., 2020). Taken together, these plant ITPKs’ biochemical literatures emphasize the notion that this enzyme family has diversified in subgroups with different biochemical function. However, this concept has not been the driver of plant ITPK research. To appreciate properly the biochemical function and physiological role of plant ITPKs, further in vitro and in vivo analyses of these enzymes are essential. This will ultimately define the plant inositol phosphate metabolic pathway in which ITPK proteins might be playing multiple and indispensable roles. Likely, the diverse and specialized ITPK enzymes are responsible to drive the cytosolic route of higher inositol phosphate synthesis (Desfougeres et al., 2019; Freed et al., 2020; Shi et al., 2003) as well as are liable to synthesize the inositol pyrophosphate IP_7_ (Adepoju et al., 2005; Laha et al., 2019).

Thanks to the ever-growing number of publicly available sequenced genomes that made our study possible. To limit the number of inaccuracy due to incomplete genomic sequence, we restricted our analyses to the reference (complete) genomes and thus proteomes. This has impeded our analysis to a tiny fraction of eukaryotes species. Furthermore, we might have missed some of the positive hits as some reference proteomes are constantly updated. Our study solidity is, nonetheless, supported by observing the loss of ITPK in many Insecta and Nematoda. This reinforces their inclusion in the Ecdysozoan clade that is still a debated issue (Telford et al., 2008).

## 4 Conclusion

The analysis of the distribution of the IP kinases across the eukaryote landscape has allowed us to observe the evolutionary themes that have shaped the IPs signalling network. Except in plant lineage, the presence of IPPK, inositol petakisphosphate 2 kinase, as a single copy is noteworthy in eukaryotes. We highlighted for the first time an IPPK loss in numerous eukaryote lineages. IPPK loss is often associated with the organism's shift to parasitism, along with a frequent and concomitant ITPK loss. This suggests the possibility of IPs acquisition by parasitic organism that are produced by the host. However, IPPK loss is also found in non-parasitic organism questioning the current view that IP_6_ is ubiquitous in eukaryotes. Alternatively, and more likely is the existence of an alternative pathway of IP_6_ synthesis.

Metazoa have expanded their IPK family members and specialized further their three identifiable catalytic activities (IP3-3Ks, IPMKs and IP6Ks) (Shears and Wang, 2019). This is contrasted with a lack of expansion of IPKs in Archaeplastida and the expansion instead of the ITPKs family. Importantly, our phylogenetic analyses suggest the differential evolution of ITPKs in plant leading to an altered enzymology somewhat comparable to the metazoan IPK family. These thoughts must foster future IP experiments.

## Funding

This work was supported by the Medical Research Council grant MR/T028904/1. D.L. further acknowledges the Deutsche Forschungsgemeinschaft (LA 4541/1-1).

## CRediT authorship contribution statement

**Debabrata Laha:** Validation, Writing - original draft, Funding acquisition. **Paloma Portela-Torres:** Investigation, Methodology, Formal analysis, Writing - review & editing, Visualization. **Yann Desfougères:** Writing - review & editing. **Adolfo Saiardi:** Conceptualization, Validation, Writing - original draft, Visualization, Funding acquisition.

## Declaration of competing interest

The authors declare none conflict of interest.

## Abbreviations

IP: inositol polyphosphate
IP6: inositol hexakisphosphate, phytic acid
IPMK: inositol phosphate multikinase
ITPK1: inositol trisphosphate I(1,3,4)P3 5/6 kinase
IPPK (IP5-2Ks): inositol pentakisphosphate I(1,3,4,5,6)P5 2-kinase
PPIP5K: diphosphoinositol pentakisphosphate kinase
IPK: inositol phosphate kinase
IP3–3K: inositol trisphosphate I(1,4,5)P3 3-Kinase
HMM: Hidden Markov Model
BLAST: Basic Local Alignment Search Tool
IPS, ISYNA1: inositol phosphate synthase
PIS, CDIPT: phosphatidylinositol synthase

Four inositol phosphate kinases families are identified based on sequence homology and structural information: ITPK1, IP_5_–2K or IPPK, PPIP5K and IPK (with different activities: IP_3_-3Ks, IPMKs, and IP6Ks). Pfam codes are given for the available family entries. For the dual domains (kinase and phosphatase) of the PPIP5K family only the Pfam entry code of the kinase domain is reported. In brackets, depicted are the *S. cerevisiae* kinase names. These kinases could catalyse the formation of both phosphoester (phosphorylation) or a high-energy phosphoanhydride bond (pyrophosphorylation). The ‘canonical’ carbon positions of the inositol ring that have been described to be phosphorylated by IPMK, ITPK1, IP_3_–3K and IPPK or IP_5_–2K or pyrophosphorylated by IP6K and PPIP5K are colour coded. In metazoans the non-kinases ISYNA1 and CDPT could be responsible for the phosphate present at position three (C3) and one (C1) respectively (dashed phosphate rings).

Loss of IPK is detected with a far lower frequency than the loss of IPPK or ITPK1. A previously described loss of ITPK1 in fungi is revealed to be less widespread than expected. Surprisingly this analysis also revealed a recurrent IPPK loss. A taxonomic group is labelled as a kinase loss if a given enzyme is absent from all reference proteomes contained within in the specific taxon. The PPIP5K family was not analysed due to the lack of an InterPro entry for the kinase domain of these dual domain proteins.

In this ‘timetree’, the length of the branches is to scale and corresponds to the evolutionary divergence of species. The different taxa were labelled according to their definition in the Open Tree of Life taxonomy database.

Phylogenetic tree, generated using the COBALT software, of the six Oryza, the four Arabidopsis, the two Marchantia ITPKs and as outsider the human ITPK1 full length proteins. This analysis reveals that plant ITPKs could be defined by at least two subgroups with higher plant ITPKs possibly differentiate in further two groups.
